# Diagnosis and subsequent US-guided percutaneous drainage of an adrenal abscess in a 5-week-old infant

**DOI:** 10.1007/s00247-012-2353-y

**Published:** 2012-04-18

**Authors:** S. C. E. Diepstraten, S. Zwaveling, F. J. A. Beek

**Affiliations:** 1 Department of Radiology, University Medical Center Utrecht, P.O. Box 85500, 3508 GA Utrecht, The Netherlands; 2 Department of Pediatric Surgery, University Medical Center Utrecht, P.O. Box 85500, 3508 GA Utrecht, The Netherlands; 3 Department of Radiology, University Medical Center Utrecht, Heidelberglaan 100, E01.132, 3584 CX Utrecht, The Netherlands

**Keywords:** Neonate, Adrenal abscess, Ultrasound, Scintigraphy

## Abstract

Adrenal abscess is an uncommon finding in neonates and young infants. It may have a fatal outcome if inadequately treated. This case report describes the successful diagnosis and treatment of a left-sided adrenal abscess in a 5-week-old girl. Abdominal US and antigranulocyte antibody-scintigraphy showed an encapsulated suprarenal mass with debris suspicious for an adrenal abscess. Treatment is generally surgical. In this case, however, we performed US-guided percutaneous drainage combined with intravenous antibiotic treatment. The child recovered fully.

## Introduction

Abscess formation in the adrenal gland is uncommon in young infants and neonates [1]. Bacterial colonisation of an adrenal haematoma is presumably the most likely cause [2]. If untreated, the condition may be lethal [2], so timely diagnosis and adequate treatment are of key importance. Surgical exploration is considered standard treatment. We present the case of a 5-week-old girl with an adrenal abscess as a complication of *Escherichia coli* sepsis. The abscess was treated successfully with US-guided percutaneous drainage.

## Case report

**Fig. 1:**
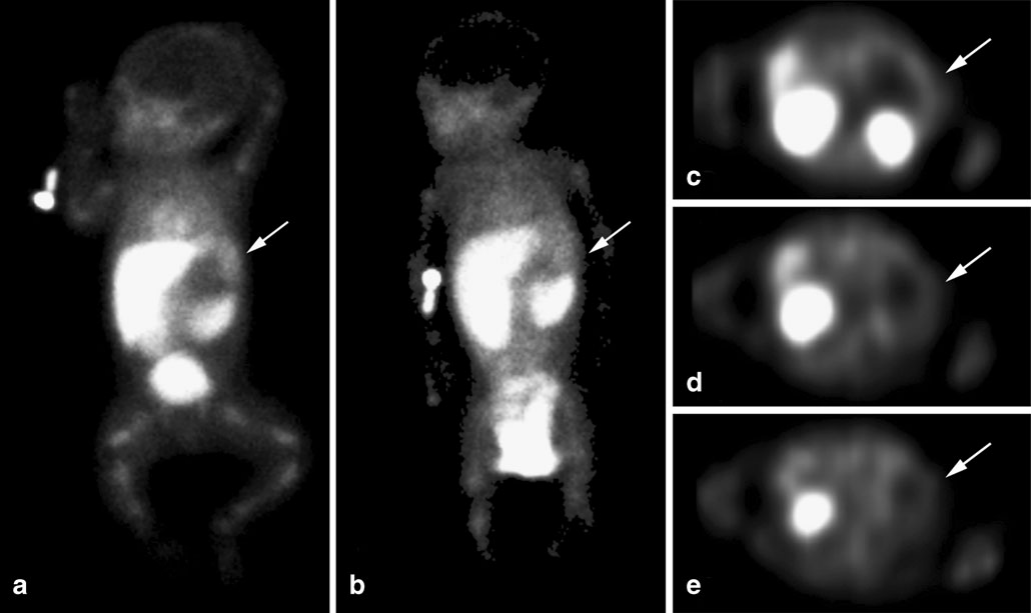
Antigranulocyte antibody-scintigraphy with 100 MBq (99 m-)Tc-sulesomab shows a photopenic area in the left upper quadrant of the abdomen surrounded by a rim of increased radioactivity uptake (*arrows*) and physiological accumulation of radioactivity in the myocardium, liver, spleen, kidneys, bladder and bone marrow. **a** Static image (anterior view) 1 h postinjection. **b** Static image (anterior view) 6 h postinjection. **c–e** SPECT-images 6 h postinjection

**Fig. 2:**
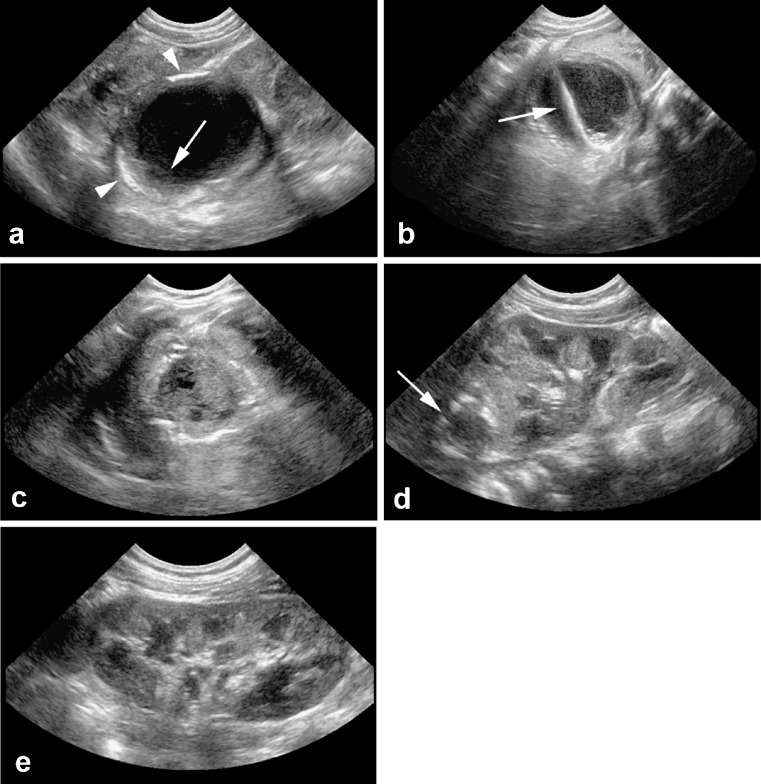
Abdominal US. **a** Images at presentation. An almost anechoic mass with some debris (*arrow*) and rim calcification (*arrowheads*) measuring 4.5 × 3.5 × 3.5 cm was seen related to the left kidney, spleen and stomach. **b** The fluid collection was drained percutaneously under US guidance with an 8-French catheter. The j-tipped guide wire (*arrow*) is visible within the collection. **c** US 6 days following drainage. The abscess has collapsed and has a largely solid aspect with some calcifications. **d** US 2 weeks following drainage. A small hypoechoic lesion (*arrow*) with rim calcification is still visible in the left adrenal region. **e** Follow-up at 4.5 months. No remaining abnormality is seen

Before referral to our department of paediatric surgery, a then 7-day-old girl presented with moaning and fever to another hospital. The girl was born at 42 + 1 weeks with an Apgar score of 9/10 and a weight of 4,412 g. The perinatal period was uneventful. On admission, physical examination revealed no abnormalities except tachypnoea. Laboratory tests showed elevated infection parameters (C-reactive protein 95 mg/l [normal range 0–10 mg/l]; white blood cell count, 15,800 μl 42^−1^ [normal range, 5,000–21,000 μl^−1^] with 11% banded neutrophils [normal range, 0–4%] and platelet count, 21,000 μl 44^−1^ [normal range, 150,000–450,000 μl^−1^]). Treatment with broad-spectrum intravenous antibiotics (gentamicin and amoxicillin/clavulanic acid) was started immediately. Several days later the blood culture proved positive for *Escherichia coli* and antibiotic treatment was switched to amoxicillin only. Cerebral fluid cultures were negative. The infection parameters and the clinical symptoms improved. After 17 days, the girl was discharged with oral antibiotics that were continued for another 2 weeks. At 17 days after discharge, during a routine follow-up, the infection parameters had again risen (C-reactive protein, 194 mg/l; white blood cell count, 29,800 with 4% banded neutrophils). There were no symptoms of infection and physical examination did not reveal an apparent focus of infection. To further assess the origin of the increased infection parameters, abdominal US was performed followed by antigranulocyte antibody-scintigraphy with 100 MBq (99 m-)Tc-sulesomab (LeukoScan; Immunomedics, New Plains, NJ). US showed a 4-cm hypoechoic mass in the left upper quadrant of the abdomen between the upper pole of the left kidney and the stomach. Scintigraphy demonstrated a photopenic area surrounded by a rim of increased radioactivity uptake in the same location, suggesting an abscess (Fig. 1). The child was subsequently referred to our hospital for further treatment. Intravenous antibiotics (gentamicin and amoxicillin/clavulanic acid) were started. Repeat abdominal US (8-5 MHz curved array transducer) confirmed a 4.4 × 3.7 × 3.5 cm hypoechoic mass anatomically related to the stomach, spleen and upper pole of the left kidney with a thickened margin, curvilinear calcifications and displacement of the left kidney. Next to this was a solid lesion with mixed echogenicity (Fig. 2). Common diagnoses such as a mesenteric cyst or an enteric duplication were considered unlikely because of the location and since the kidney was compressed by the mass. To assess the probability of a neuroblastoma, laboratory tests for catecholamines were performed, which were normal. It was concluded that the imaging finding were consistent with an adrenal haemorrhage complicated by secondary infection and abscess formation.

The mass was drained percutaneously under US guidance with an 8-French catheter and pus was retrieved. Additionally, a central venous catheter was placed for antibiotic treatment. As expected, subsequent bacteriological cultures from the pus were positive for *E. coli* and antibiotic therapy was changed to amoxicillin only. The drain remained in situ until pus production had ceased 3 days later. Laboratory tests showed normalising infection parameters. At 6 days after drainage, abdominal US showed a residual mass measuring 2.5 × 1.7 cm and minimal compression of the upper pole of the left kidney. After another day, the central venous catheter had to be removed due to infection and oral administration of amoxicillin was started. The girl was discharged 2 days later. Antibiotics were ceased 10 days after drainage and further recovery was uneventful. At 5-months follow-up, no complications had occurred and abdominal US showed a normal left adrenal gland.

## Discussion

This case describes the successful diagnosis and treatment of a neonatal adrenal abscess. An hypoechoic abdominal lesion with a thick, calcified rim and central debris was seen sonographically. The differential diagnosis of such cystic adrenal masses is wide, including a variety of congenital deformations and neo-/dysplasias [3], of which neuroblastoma is the most common. The clinical presentation and course in our case were strongly suggestive of an infectious condition. Scintigraphy supported this notion. Due to the location of the cystic mass inferior to the stomach and spleen, the compression/displacement of the kidney and the absence of dilatation of the renal collecting system, the lesion was deemed most likely to originate from the adrenal gland.

Differentiating between a (cystic) neuroblastoma and an adrenal abscess is challenging [4]. Although elevated urinary catecholamines are strongly suggestive of neuroblastoma, normal levels are not sufficient to exclude it [4]. Additional sonographic features made neuroblastoma unlikely in our case. Calcifications and haemorrhage may be seen in neuroblastoma, but calcifications are usually stippled rather than curvilinear as in our patient [1, 3]. Neuroblastoma in the newborn has a good prognosis. It often regresses spontaneously in children younger than 1 year of age [5]. We therefore felt that short-term follow-up by US was an appropriate strategy to rule out neuroblastoma and chose to refrain from more invasive diagnostic procedures such as abscess wall biopsy or surgical resection. US at 5 months’ follow-up showed complete regression of the lesion.

In most previous case reports, neonatal adrenal abscesses were treated with surgical exploration and drainage, which have been recommended as the therapies of choice for larger lesions [2]. Due to technical developments, less invasive procedures may increasingly suffice. In our case, the abscess could be drained percutaneously with an 8-French catheter under US-guidance, and no complications occurred during follow-up. Previous cases where US-guided percutaneous drainage was used were reported by Mondor et al. [6] and Chin-Theam et al. [7]. The former performed an uncomplicated percutaneous US-guided drainage of a right-sided adrenal abscess measuring 6 × 8 cm using a 6-French pigtail catheter by a posterolateral approach. The latter performed US-guided aspiration of bilateral adrenal masses measuring 5 × 7 cm and 3 × 3 cm with a 21-G needle by a posterior approach. In both cases, treatment resulted in complete recovery.

Sonography is advocated as the first-line diagnostic imaging modality for evaluating abdominal masses in children. It is believed to be the most useful modality to define the morphology of the mass and to determine the relation of the mass to the kidney [2]. In our case, an additional antigranulocyte antibody-scintigraphy with (99 m-)Tc-sulesomab was performed to identify potential sources of infection. Leukoscan was developed initially for diagnostic purposes in patients with suspected bone or joint infections, but has also been shown to be useful for diagnosis of soft tissue infections [8]. Scintigraphy was helpful by confirming a probable abscess in the adrenal region and ruling out other foci of infection.

In conclusion, we have reported the successful treatment of a neonatal adrenal abscess with US-guided percutaneous drainage, a technique not performed routinely. In our opinion, this less invasive procedure may be considered as an alternative to surgical exploration when imaging is highly suggestive of an adrenal abscess. Our report also confirms that an adrenal abscess in a neonate can be assessed by antigranulocyte antibody-scintigraphy.
